# Effectiveness of temocillin in treatment of non-urinary tract infections caused by ESBL-producing Enterobacterales and risk factors for failure

**DOI:** 10.1093/jacamr/dlae164

**Published:** 2024-10-17

**Authors:** Christel Mamona Kilu, Camille Menvielle, Anne Cataldi, Antoine Hamon, Clara Duran, Cedric Mwanba, Chloé Tesmoingt, Laura Bouabdallah-Perrin, Pauline Touche, Aurélie Chanh Hew Wai, Clément Ourghanlian, Marie Antignac, Marc-Antoine Bildan, Alexandre Bleibtreu, Hugues Michelon, Sylvain Diamantis, Benoit Pilmis, Antoine Citerne, Eric Farfour, Aurélien Dinh, Paris Temocillin study group, Aurélien Dinh, Christel Mamona, Clara Duran, Hugues Michelon, Frédérique Bouchand, Eric Farfour, Pauline Touche, Aurélie Chan Hew Wai, Benoît Pilmis, Sylvain Diamantis, Rui Batista, Etienne Canouï, Antoine Citerne, Laurène Deconinck, Chloé Tesmoingt, Laura Bouabdallah Perrin, Matthieu Lafaurie, Sophie Touratier, Victoire de Lastours, Antoine Hamon, Marie Antignac, Céline Leplay, Jean-Luc Meynard, Anne Cataldi, Clément Ourghanlian, Raphaël Lepeule, Marc-Antoine Bildan, Marie-Caroline Loustalot, Ruxandra Calin, Cédric Mwamba, Jean Baptiste Pain, Lelia Escaut, Benjamin Wyplosz, Alexandre Bleibtreu, Helga Junot

**Affiliations:** Infectious Disease Department, Raymond Poincaré Hospital, APHP, Garches, France; Infectious Disease Department, Pitié-Salpêtrière Hospital, APHP, Paris, France; Pharmacy, Henri Mondor Hospital, APHP, Créteil, France; Internal Medicine, Beaujon Hospital, APHP, Clichy, France; Infectious Disease Department, Raymond Poincaré Hospital, APHP, Garches, France; Pharmacy, Tenon Hospital, APHP, Paris, France; Pharmacy, Bichat Hospital, APHP, Paris, France; Pharmacy, Saint-Louis Hospital, APHP, Paris, France; Pharmacy, Foch Hospital, Suresnes, France; Pharmacy, Foch Hospital, Suresnes, France; Pharmacy, Henri Mondor Hospital, APHP, Créteil, France; Pharmacy, Saint-Antoine Hospital, APHP, Paris, France; Pharmacy, Européen Georges Pompidou Hospital, APHP, Paris, France; Infectious Disease Department, Pitié-Salpêtrière Hospital, APHP, Paris, France; Infectious Disease Department, Raymond Poincaré Hospital, APHP, Garches, France; Infectious Disease Department, Melun Hospital, Melun, France; Infectious Disease Department, Saint-Joseph & Marie-Lannelongue Hospital, Paris, France; Pharmacy, Cochin Hospital, APHP, Paris, France; Pharmacy, Foch Hospital, Suresnes, France; Infectious Disease Department, Raymond Poincaré Hospital, APHP, Garches, France; IHU PROMETHEUS, Raymond Poincaré Hospital, APHP, Garches, France

## Abstract

**Objectives:**

To describe the real-life use of temocillin for non-urinary tract infections, to assess its effectiveness in infections caused by ESBL-producing Enterobacterales, and to identify risk factors for treatment failure.

**Method:**

Retrospective multicentric study in 14 tertiary care hospitals, including all patients who received at least one dose of temocillin for ESBL infections from 1 January 2016 to 31 December 2021 for non-urinary tract infections. Failure was a composite criterion defined within 28 day follow-up by persistence or reappearance of signs of infection, and/or switch to suppressive antibiotic treatment and/or death from infection. Logistic regression with univariable and multivariable analysis was performed to identify risks associated with failure.

**Results:**

Data on 163 infection episodes were collected; 133 were due to ESBL-producing Enterobacterales and 128 were included in the effectiveness analysis. Median (IQR) age was 61 (53–70) years and 61.7% of patients were male. Main indications were lower respiratory tract infection (LRTI; 28.9%), intra-abdominal infections (IAI; 28.1%) and cutaneous infections (12.5%). The main bacteria involved were *Klebsiella pneumoniae* (48.4%), *Escherichia coli* (25.0%) and *Enterobacter cloacae* (24.2%). Polymicrobial infections occurred in 45.3% of cases. Temocillin was used as monotherapy in 86/128 (67.2%). Failure was found in 36/128 (28.1%) cases. In multivariable analysis, the only factor associated with failure was initial severity of the episode [adjusted OR 3.0 (95% CI: 1.06–8.69)].

**Conclusions:**

During non-urinary tract infections, the main use of temocillin was for LRTIs and IAIs due to ESBL-producing *E. coli* and *K. pneumoniae*. The main risk factor for failure was initial severity of the disease.

## Introduction

ESBL-producing Enterobacterales (ESBL-E) have spread worldwide and could be responsible for difficult-to-treat infection.^1–3^ Carbapenems are often used as first-line antibiotics against infections caused by ESBL-E.^4,5^ The increased use of carbapenems may lead to rising resistance to carbapenems among Gram-negative bacteria, which is associated with high costs and high mortality.^6–8^ For that reason, it is essential to consider non-carbapenem antibiotics.

Temocillin, a semisynthetic 6-α-methoxylpenicillin antibiotic derived from ticarcillin, has demonstrated stability against ESBL-, AmpC- and some KPC-producing Gram-negative bacteria.^9–11^ Temocillin has no activity against Gram-positive bacteria, anaerobes or non-fermenters like *Pseudomonas aeruginosa* and *Acinetobacter baumanii*.^12^ This narrow spectrum has minimized selection pressure on microbiota.^13,14^

Temocillin is used mainly for carbapenem-sparing in urinary tract infections (UTIs) with high clinical success rate.^15–17^ However, its use and efficacy for non-urinary tract infections is not well known.

We describe the use of temocillin in real-life settings and evaluate its effectiveness in treating non-urinary tract infections. Additionally, we aim to identify risk factors associated with treatment failure.

## Materials and methods

A multicentre retrospective study was conducted in the greater Paris area, including all adult patients who received temocillin for non-urinary tract infections for at least 1 day from 1 January 2016 to 31 December 2021.

Data were collected from medical charts in 14 tertiary care hospitals. We collected demographic characteristics (age, sex, comorbidities, risk factors etc.), clinical, biological and microbiological data (clinical and severity signs, laboratory tests, organisms identified), therapeutic data (dosage of temocillin, other associated antibiotics), and adverse events and clinical outcome at Day 28 of the first temocillin dose and at the patient’s last visit.

Immunosuppression was defined as the presence of the following criteria: asplenia, neutropenia, agammaglobulinaemia, organ transplant, haematological malignancies, HIV infection with CD4 cell count below 200 cells/mm^3^, 20 mg of prednisolone equivalent over at least 3weeks, cancer chemotherapy or other immunosuppressive drugs (e.g. cyclophosphamide, azathioprine, cyclosporine etc.).

Neurological disease was defined as the presence of the following criteria: cerebral vascular disease, spinal cord injury, multiple sclerosis or Parkinson’s disease.

Bacterial strain and resistance mechanism (ESBL, AmpC) analyses and antibiotic susceptibility testing were performed using disc diffusion, and MICs were determined by broth microdilution, in the local laboratories of the centres, according to EUCAST and CLSI guidelines.^18^

Severe infection was defined as the need for hospitalization in an ICU. Failure was a composite criterion defined within a 28 day follow-up period by persistence or reappearance of signs of infection, and/or death from infection. Patients with missing data at Day 28 were excluded from this analysis.

Quantitative variables are presented as median (IQR), while qualitative variables are presented as number of occurrences and percentage. Enterobacterales were speciated and grouped as AmpC, ESBL or non-AmpC/non ESBL based on resistance patterns. Antibiotics received before temocillin were reviewed and analysed.

The distribution of categorial variables were compared using chi-squared tests; *t*-tests were used to compare the distribution of quantitative variables. A *P* value of <0.05 was considered statistically significant.

To identify risk factors associated with failure, a univariable analysis by logistic regression was performed, using demographic and medical characteristics, as well as all clinical and biological data. For patients requiring renal dosage adjustments, temocillin dosage used in the statistical analyses was the targeted dosage before reduction. A multivariable analysis by logistic regression was then performed using all variables from the univariable analysis that had a *P* value of <0.05. ORs were calculated from the univariate and multivariable analysis to quantify association with failure at Day 28 with 95% CIs.

All analyses were performed with R statistical software, version 4.3.1.

### Ethics

Considering the retrospective study design, data collection from pre-existing medical records, and respect for the anonymity of the patients included (referred to as studies ‘Hors Loi Jardé’ in France), no ethical approval or administrative approval were necessary for this study. This study was approved by the French Data Protection Agency (CNIL), with the number 2225429 v 0.

## Results

### Baseline description

**Figure 1.**
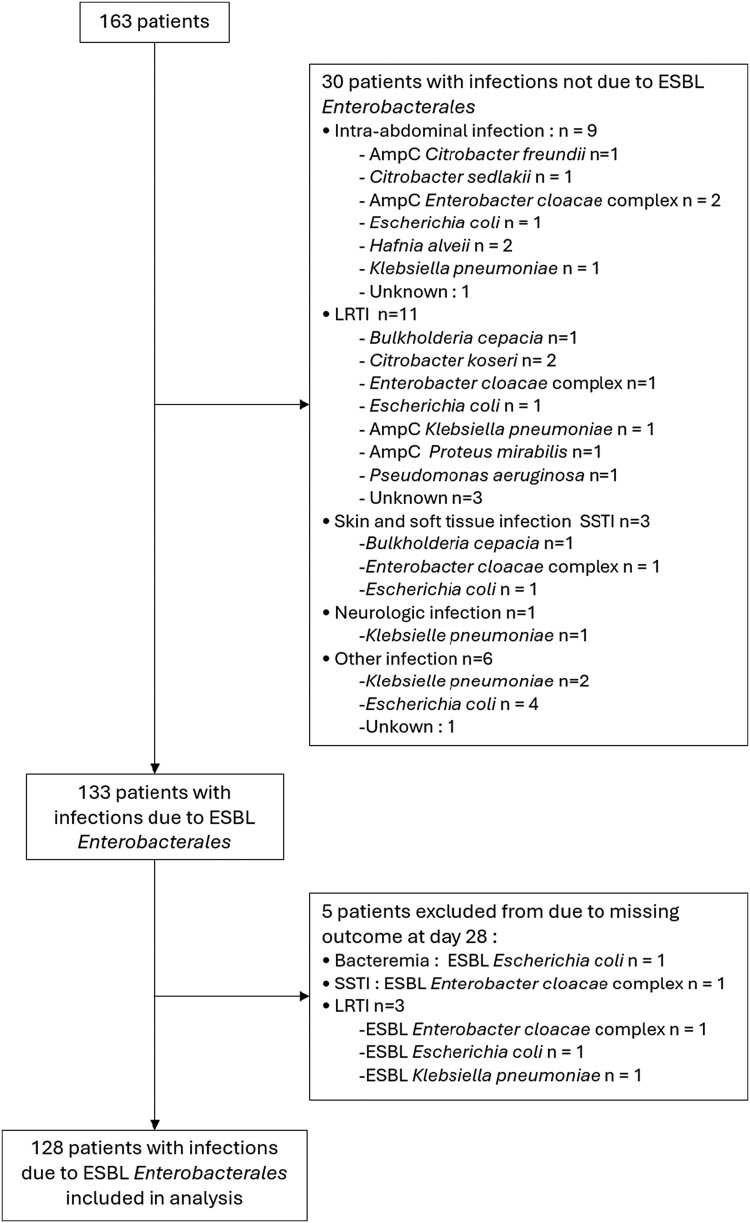
Flow chart.

After exclusion of prophylactic treatment, 163 patients were treated with temocillin, and 133 of them presented with ESBL-producing Enterobacterales (ESBL-E) infections. Five patients were excluded from the effectiveness analysis due to missing data at Day 28 (see flow chart presented in Figure 1).

Male patients represented 61.7% (79/128) of the total study population. The median (IQR) age was 61 (53–70) years. The main sources of infections were lower respiratory tract infections (LRTIs), representing 28.9% (*n* = 37/128) of patients, followed by intra-abdominal infections (IAIs), representing 28.1% (*n* = 36/128). Forty-four patients (34.4%) presented with severe infection requiring hospitalization in an ICU (Table 1).

**Table 1.** Population characteristics of patients treated with temocillin for non-urinary tract infections

	Total *N* = 128	Cure group *N* = 92	Failure group *N* = 36	*P* value
Age (years), median (IQR)	61 (53–70)	61 )54–69)	63 (52–71)	0.5
Male patient, *n* (%)	79 (61.7)	54 (58.7)	25 (69.4)	0.3
Hospital ward, *n* (%)				
ICU	44 (34.4)	23 (25.0)	21 (58.3)	<0.001
Medicine	62 (48.4)	50 (54.3)	12 (33.3)	0.032
Surgery	25 (19.5)	22 (23.9)	2 (8.3)	0.046
Comorbidities, *n* (%)				
Chronic respiratory failure	9 (7.0)	7 (7.6)	2 (5.6)	>0.9
Heart disease	31 (24.2)	20 (21.7)	11 (30.6)	0.3
Chronic renal failure	26 (20.3)	19 (20.6)	7 (19.4)	0.9
Liver failure	20 (15.6)	15 (16.3)	5 (13.9)	0.7
Neurological disease	13 (10.2)	9 (9.8)	4 (11.1)	0.8
Immunodepression	52 (40.6)	36 (39.1)	16 (44.4)	0.6
AIDS	6 (4.7)	3 (3.3)	3 (8.3)	0.4
Neutropenia <500 cells/mm^3^	2 (1.6)	2 (2.2)	0 (0.0)	>0.9
Chemotherapy	22 (17.5)	16 (17.8)	6 (16.7)	0.9
Immunosuppressive	24 (18.8)	17 (18.5)	7 (19.4)	0.9
Corticosteroids >20 mg/L	12 (9.4)	8 (8.8)	4 (11.1)	0.7
Diabetes mellitus	39 (30.7)	29 (31.9)	10 (27.8)	0.7
Renal clearance (mL/min), median (IQR)	82 (52–136)	86 (54–141)	75 (49–117)	0.5
Site of infection, *n* (%)				
LRTI	37 (28.9)	20 (21.7)	17 (47.2)	0.004
IAI	36 (28.1)	30 (32.6)	6 (16.7)	0.071
Intravascular device infection	19 (14.8)	15 (16.3)	4 (11.1)	0.3
Skin and soft tissue infection	16 (12.5)	13 (14.1)	3 (8.3)	0.6
Bone and joint infection	7 (5.5)	7 (7.7)	0 (0.0)	0.2
Bloodstream infection	4 (3.1)	3 (3.3)	1 (2.8)	>0.9
Other type of infection^a^	3 (2.4)	1 (1.1)	2 (5.5)	>0.9
Severity, *n* (%)	46 (35.9)	25 (27.2)	21 (58.3)	<0.001
Before temocillin treatment				
Surgical treatment, *n* (%)	42 (32.8)	35 (38.0)	4 (19.4)	0.044
Number of antibiotic treatment lines, median (IQR)	1 (1–2)	1 (1–2)	2 (1–2)	0.5
Microbiology analysis, *n* (%)				
Polymicrobial infection	58 (46.0)	42 (42.2)	16 (45.7)	>0.9
ESBL-producing *E. coli*	32 (25.0)	21 (22.8)	11 (30.6)	
ESBL-producing *Enterobacter cloacae* complex	31 (24.2)	23 (25.0)	8 (22.2)	
ESBL-producing *K. pneumoniae*	62 (48.4)	46 (50)	16 (44.4)	
Temocillin dosage, *n* (%)				0.7
Less than 4 g per day or equivalent^b^	11 (9.3)	7 (7.9)	4 (13.8)	
4 g per day or equivalent^b^	32 (27.1)	25 (28.1)	7 (24.1)	
At least 6 g per day or equivalent^b^	75 (63.6)	57 (64.0)	18 (62.1%)	
Treatment duration (days), median (IQR)	7 (4–11)	7 (4–13)	7 (4–9)	0.8
Associated antibiotic, *n* (%)	42 (33.1)	30 (33.0)	12 (33.3)	0.7

^a^Surgical site infection (*n* = 2), wall abscess (*n* = 1).

^b^According to renal function.

Fifty-eight (46.0%) infections were polymicrobial and ESBL-producing *Klebsiella pneumoniae* was the most frequently isolated bacteria (48.4%; *n* = 62/128). Bacteria involved according to source are presented in Table 2. Prior to temocillin, 52 patients (40.6%) received carbapenems and 29 (22.6%) received piperacillin/tazobactam. Six patients (4.7%) received temocillin as first-line empirical treatment.

**Table 2.** Microbiological results depending on type of infection

Type of infection	*n*
LRTI	37
Monomicrobial	16
ESBL-producing *E. coli*	6
ESBL-producing *K. pneumoniae*	8
ESBL-producing *Klebsiella aerogenes*	1
ESBL-producing *Klebsiella oxytoca*	1
Polymicrobial	21
ESBL-producing *E. coli *+ non-ESBL-producing *Serratia marcescens*	1
ESBL-producing *E. coli *+ Gram positive bacteria	2
ESBL-producing *K. pneumoniae* + non-ESBL-producing *E. coli*	1
ESBL-producing *K. pneumoniae *+* *non-ESBL-producing *E. cloacae* complex	2
ESBL-producing *K. pneumoniae *+* A. baumannii*	2
ESBL-producing *K. pneumoniae *+* *Gram-positive bacteria	4
ESBL-producing *E. cloacae* complex + *Candida albicans*	1
ESBL-producing *E. cloacae* complex + Gram-positive bacteria	4
ESBL-producing *E. cloacae* complex +* *ESBL-producing *K. oxytoca*	1
ESBL-producing *E. cloacae* complex +* P. aeruginosa*	2
ESBL-producing *E. cloacae* complex + non-ESBL-producing *Citrobacter koseri*	1
IAI	35
Monomicrobial	23
ESBL-producing *E. coli*	7
ESBL-producing *E. cloacae* complex	7
ESBL-producing *K. pneumoniae*	9
Polymicrobial	12
ESBL-producing *E. coli *+ ESBL-producing *K. pneumoniae*	1
ESBL-producing *E. coli *+* *non-ESBL-producing *K. pneumoniae*	1
ESBL-producing *E. coli *+ ESBL-producing *E. cloacae* complex	1
ESBL-producing *E. coli *+* Weissella confusa*	1
ESBL-producing *E. coli + *AmpC-producing *Enterobacter kobei*	1
ESBL-producing *E. coli *+ Gram-positive bacteria	2
ESBL-producing *K. pneumoniae* + non-ESBL-producing *E. coli*	3
ESBL-producing *K. pneumoniae* + non-ESBL-producing *K. aerogenes*	1
ESBL-producing *K. pneumoniae* + AmpC-producing *Hafnia alvei*	1
Skin and soft tissue infection	16
Monomicrobial	9
ESBL-producing *E. coli*	1
ESBL-producing *K. pneumoniae*	5
ESBL-producing *E. cloacae*	3
Polymicrobial	7
ESBL-producing *C. koseri *+* *non-ESBL-producing *Proteus mirabilis*	1
ESBL-producing *K. pneumoniae* + ESBL-producing *E. cloacae*	2
ESBL-producing *K. pneumoniae *+ non-ESBL-producing *P. mirabilis*	1
ESBL-producing *K. pneumoniae *+ Gram-positive bacteria	2
ESBL-producing *K. pneumoniae *+* Stenotrophomonas maltophilia*	1
Bone and joint infection	7
Monomicrobial	4
ESBL-producing *K. pneumoniae*	3
ESBL-producing *E. cloacae*	1
Polymicrobial	3
ESBL-producing *E. cloacae *+ Gram-positive bacteria	1
ESBL-producing *K. pneumoniae *+ Gram-positive bacteria	2
Other infections	25
Monomicrobial	13
ESBL-producing *E. coli*	1
ESBL-producing *K. pneumoniae*	6
ESBL-producing *E. cloacae*	6
Polymicrobial	12
ESBL-producing *E. coli *+* *non-ESBL-producing *P. mirabilis*	1
ESBL-producing *E. coli *+* P. aeruginosa*	1
ESBL-producing *E. coli + *ESBL-producing *K. pneumoniae*	1
ESBL-producing *K. pneumoniae *+* *non-ESBL-producing *E. coli*	2
ESBL-producing *K. pneumoniae *+* *non-ESBL-producing *P. mirabilis*	1
ESBL-producing *K. pneumoniae + S. maltophilia*	1
ESBL-producing *E. cloacae *+* *non-ESBL-producing *K. pneumoniae*	1
ESBL-producing *E. cloacae *+ Gram-positive bacteria	4

Median (IQR) duration of treatment was 7 (4–11) days. Seventy-five patients (63.6%) received 6 g of temocillin, associated in 42 cases (33.1%) with other antibiotics such as an anti-Gram-positive antibiotic (*n* = 12/128).

### Follow-up at 28 days

Overall, failure was observed in 33 patients (28.1%) at Day 28.

Among patients treated with a temocillin dosage of ≥6 g per day, 57/75 (76.0%) had a favourable outcome at Day 28. Among these, 27/75 (36.0%) presented with LRTIs.

Among patients treated with a temocillin dosage lower than 6 g per day, 32/43 (74.4%) had a favourable outcome at Day 28. Among these, 11/43 (25.6%) presented with LRTIs.

In the multivariable analysis, the only risk factor for failure was severity of infection [adjusted OR (aOR): 3.0; 95% CI: 1.06–8.69; *P* = 0.039) (Table 3). Although not significant in multivariable analysis, the risk of failure tended to be high in LRTIs.

**Table 3.** Multivariable regression factors associated with failure for 128 patients treated with temocillin for non-urinary tract infections

	Univariable		Multivariable	
	OR (95% CI)	*P* value	OR (95% CI)	*P* value
Severe infection (ICU)	4.81 (2.15–11.3)	<0.001	3.0 (1.06–8.69)	0.039
Hospitalization in medical ward	0.42 (0.18–0.93)	0.035	0.61 (0.21–1.73)	0.3
Hospitalization in surgical ward	0.29 (0.07–0.91)	0.057		
LRTI	3.22 (1.42–7.39)	0.005	1.26 (0.44–3.48)	0.7
Surgical treatment before temocillin	0.39 (0.15–0.95)	0.048	0.46 (0.15–1.32)	0.2

Comorbidities, bacterial species and the dosage of temocillin seem to have had no impact on the outcome in the descriptive analysis (Table 1).

### Adverse events

Only two *Clostridioides difficile* infections and one case of acute renal injury were reported as potential temocillin-related adverse events. No serious adverse drug reactions leading to discontinuation were observed.

## Discussion

We conducted a large, multicentre cohort study to evaluate the real-life use of temocillin and its effectiveness in treating non-urinary tract infections caused by ESBL-E.

Temocillin is approved in Europe for treating bacteraemia, UTI and LRTI at a dosage of 2 g twice daily. It is currently available only in the UK, Belgium, Germany and France.^9,15,19–22^ Temocillin is particularly relevant for treating infections caused by resistant Gram-negative strains. A recent study found a 61.8% susceptibility rate among 400 isolates, including those producing ESBL, AmpC and KPC, using the BSAC breakpoint for systemic infections (≤8 mg/L).^23^

Our study confirmed temocillin’s effectiveness for treating non-urinary tract infections caused by ESBL-E. Furthermore, the study population frequently included patients with severe infections and many were immunocompromised. The main indications were LRTI and IAI, types of infections with limited data on temocillin’s effectiveness. Alexandre *et al*.^24^ found significantly lower clinical failure rates for UTIs compared with non-urinary tract infections (4.9% versus 26.7%; *P* = 0.001). Notably, clinical failure rates were significantly different between sepsis and severe sepsis or septic shock treated with temocillin (6.2% versus 25%; *P* = 0.011), although no significant variations were noted between different dosages or bacteria. These results are consistent with our data.

For LRTIs, clinical data on temocillin for both community-acquired and hospital-acquired pneumonia are limited, and information on epithelial lining fluid (ELF)/plasma penetration ratios is sparse.^25,26^ Temocillin is minimally effective against Gram-positive microorganisms and certain Gram-negative non-fermenters such as *A. baumannii* and *P. aeruginosa*. *In vitro* studies suggest that combination regimens may enhance its activity.^27,28^ In our study, about one-third of patients with LRTIs (12/37) received combination therapy with antibiotics like linezolid, vancomycin, quinolones, ceftazidime or trimethoprim/sulfamethoxazole.

A retrospective audit by Habayeb *et al*.^29^ compared piperacillin/tazobactam with amoxicillin plus temocillin in 192 cases of hospital-acquired pneumonia, and found no difference in clinical success rates between the two groups. However, patients treated with amoxicillin plus temocillin experienced significantly fewer episodes of diarrhoea and *C. difficile* infection.

In a study testing continuous infusion of 4 g per day of temocillin in ICU patients with nosocomial pneumonia, the drug maintained stability for 24 h and was compatible with concurrent administration of flucloxacillin and aminoglycosides. Despite achieving stable free serum concentrations above the breakpoint of 16 mg/L, the authors recommended considering a lower breakpoint of 8 mg/L due to individual variations in this population.^30^

For ventilator-associated pneumonia (VAP), temocillin is often administered via continuous infusion of 6 g daily, according to recent studies.^31^ Most patients in our study also received continuous or prolonged infusions of 6 g of temocillin daily.

For IAIs, pharmacokinetic/pharmacodynamic studies suggest that temocillin penetrates bile and peritoneal fluid at approximately 80%. Small case series have reported high clinical cure rates.^17,19,32^ In our study, more than a quarter of patients had IAI, which resulted in one of the highest success rates, even though it is not currently included in national guidelines.

A study on patients with peritonitis and intra-abdominal abscesses demonstrated the effectiveness of 1 g temocillin twice daily. This study included a broad range of bacteria susceptible to temocillin and found favourable outcomes in most cases, with no reported adverse reactions.^33^

In the peritoneal setting, temocillin rapidly penetrated, achieving a mean peritoneal concentration of 49.1 mg/L. This suggests that 1 g of temocillin administered twice daily reaches sufficiently high intraperitoneal levels to inhibit susceptible pathogens.^34^ Wittke *et al*.^35^ reported that a dosage of 2 g temocillin twice daily during biliary surgery showed good clinical effectiveness with few side effects. Regarding MDR microorganisms, Alexandre *et al*.^19^ demonstrated significant temocillin activity in peritoneal fluid, blood and spleen in a murine model infected with KPC-producing *Escherichia coli*. Furthermore, most patients with IAI in our study underwent surgery, contributing to the high rate of favourable outcomes in our cohort.

For bloodstream infections (BSIs), a study of 92 patients treated with temocillin for bacteraemia due to Enterobacterales reported an 84% cure rate, although no comparative studies with carbapenems have been published to our knowledge.^36^ The optimal dosage for this indication remains debated, with higher cure rates noted for 2 g twice daily versus less than 2 g twice daily, especially in the ESBL or derepressed AmpC subset.^36^

Regarding bone and joint infections and skin and soft tissue infections, data are currently limited. A single case report highlights temocillin’s potential in managing peripheral phlebitis associated with a *K. pneumoniae* infection complicating a psoas abscess caused by *Staphylococcus aureus*.^37^ Similarly, there is a paucity of studies on temocillin in osteoarticular contexts. Specifically, two cases documented its use in knee arthritis induced by *Pantoea agglomerans* and cervical osteomyelitis caused by *Burkholderia cepacia*.^38,39^ Additionally, some authors suggest to incorporate temocillin into antibiotic-loaded.^40^

In our study, temocillin was primarily used as a switch from first-line carbapenems, serving as a carbapenem-sparing treatment. There were few empirical uses in accordance with French guidelines due to its limited activity against Gram-positive bacteria and anaerobes. No significant differences in success rates were observed based on the causative microorganism. Few adverse events were reported, with only two cases of *C. difficile* infection, confirming temocillin’s safety.

### Limitations

This study has several limitations. As a retrospective study, it is subject to potential selection bias and confounding factors. Additionally, no control group was included. We were also unable to provide detailed information regarding source control or the distribution of continuous versus intermittent temocillin administration. Most patients received carbapenem antibiotics before switching to temocillin, and approximately 33% received an additional antibiotic with temocillin, which introduces bias in evaluating temocillin’s efficacy but still reflects real-world use of the drug.

### Conclusions

The primary use of temocillin for non-urinary tract infections was in the treatment of LRTIs caused by ESBL-producing *K. pneumoniae*. The main risk factor for treatment failure was the initial severity of the infections. Temocillin appears to be an effective option for treating non-urinary tract infections caused by susceptible pathogens and may serve as a viable alternative to carbapenems for eradicating ESBL-E. Its advantages include a favourable safety profile and a low incidence of adverse events. However, ongoing debates about clinical breakpoints and optimal dosages highlight the need for further clinical studies to better define its role and efficacy.
